# Editorial Comment: Dietary and circulating vitamin D and risk of renal cell carcinoma: a meta-analysis of observational studies

**DOI:** 10.1590/S1677-5538.IBJU.2020.0417.1

**Published:** 2021-07-20

**Authors:** Bianca Martins Gregorio

**Affiliations:** 1 Universidade do Estado do Rio de Janeiro - UERJ Unidade de Pesquisa Urogenital Rio de JaneiroRJ Brasil Unidade de Pesquisa Urogenital, Universidade do Estado do Rio de Janeiro - UERJ, Rio de Janeiro, RJ, Brasil

## COMMENT

Doctor Wu and colleagues from China conducted an important meta-analysis of observational studies on dietary and circulating levels of vitamin D and its relationship to the risk of renal cell carcinoma (RCC) (1). The authors based their scientific research on well-known international databases from inception through February 2019. They observed that both the level of circulating vitamin D and the increase in dietary intake of the same vitamin may be associated with reduced risk of RCC.

Malignant kidney tumors are increasingly on the rise. In the USA, in 2018, 63,000 new cases emerged and about 15,000 individuals died from kidney cancer, in addition to a quantitative of 350,000 new cases of the disease worldwide. Among the various histological subtypes of renal cancer, RCC is the most common (75% of cases) (2), with a high incidence of recurrence (30-40%) and the development of metastatic disease (10%) for the lung, bone, brain, liver and adrenal glands during an interstitium of five years (3). The etiology is still unknown, but it is believed that some genetic polymorphisms increase the susceptibility to the development of the disease (4).

The literature describes that the Vitamin D nuclear receptor (VDR), which controls vitamin D activity, could influence the expression of hundreds of genes,

including crucial genes in cell-cycle regulation, differentiation, and cancer pathology, being therefore greatly affected by these genetic polymorphisms (5, 6). In addition, ideal vitamin D stores in the body are also important since low serum concentrations of 25-hydroxyvitamin D (25 [OH] D) (form of primary vitamin D storage) have been associated with increased morbidity and mortality for cardiovascular disease and cancer (6).

Vitamin D (“bone vitamin”) is a hormone synthesized in the skin through an isomerization reaction catalyzed by type B ultraviolet radiation (UV-B), in which the active form regulates the metabolism of calcium and phosphorus (7). The vitamin D nutritional recommendation, in relation to dietary consumption, are stipulated by the Daily Recommendation (Recommended Dietary Allowances - RDA) (8). Although few studies relate dietary vitamin D to health measures, most guidelines state that serum 25 (OH) D concentration <12 ng / mL indicates frank deficiency and 12 - 20 ng / mL is insufficient for optimal bone health. However, establishing an optimal serum 25 (OH) D concentration has proven more controversial. The Institute of Medicine (IOM) guidelines chose 20 ng / mL as sufficient for 97.5% of the population (8) while the Endocrine Society classified> 30 ng / mL as satisfactory and 20 - 30 ng / mL as insufficient (9). For practical and biological purposes, the IOM recommends a daily intake of 600 IU (40 IU = 1 µg) for individuals between 1 and 70 years old and 800 IU for individuals over 71 years old (8), remembering that the main dietary sources are fish from cold water, such as salmon and tuna, and vegetable sources such as vegetable oils (10).

In summary, correlating dietary vitamin D intake to circulating levels of the same vitamin is essential and can be associated with reduced risk of RCC (1). Maintaining healthy eating habits with regular exposure to sunlight can represent the way to improve vitamin D status and promote kidney health. However, more studies are needed to confirm the data obtained with this meta-analysis.
